# Using RosettaLigand for Small Molecule Docking into Comparative Models

**DOI:** 10.1371/journal.pone.0050769

**Published:** 2012-12-11

**Authors:** Kristian W. Kaufmann, Jens Meiler

**Affiliations:** 1 Department of Chemistry, Vanderbilt University, Nashville, Tennessee, United States of America; 2 Department of Pharmacology, Vanderbilt University Medical Center, Nashville, Tennessee, United States of America; 3 Center for Structural Biology, Vanderbilt University, Nashville, Tennessee, United States of America; 4 Institute of Chemical Biology, Vanderbilt University, Nashville, Tennessee, United States of America; University of Rome, Italy

## Abstract

Computational small molecule docking into comparative models of proteins is widely used to query protein function and in the development of small molecule therapeutics. We benchmark RosettaLigand docking into comparative models for nine proteins built during CASP8 that contain ligands. We supplement the study with 21 additional protein/ligand complexes to cover a wider space of chemotypes. During a full docking run in 21 of the 30 cases, RosettaLigand successfully found a native-like binding mode among the top ten scoring binding modes. From the benchmark cases we find that careful template selection based on ligand occupancy provides the best chance of success while overall sequence identity between template and target do not appear to improve results. We also find that binding energy normalized by atom number is often less than −0.4 in native-like binding modes.

## Introduction

Structure based comparative models of proteins in complex with small molecules advance science by creating hypotheses that can be tested experimentally. The process of modeling a protein in complex with a small molecule, often termed small molecule docking, has a long history stretching back more than 25 years [1]. There are two basic problems in small molecule docking, searching the space of possible arrangements of the atoms at the small molecule protein interface (sampling) and evaluating free energy of the binding pose (scoring). Sampling requires accounting for both the position of the small molecule relative to the protein as well as the internal flexibility of the small molecule and the protein. This easily leads to thousands of degrees of freedom. In order to detect the correct binding pose the method must accurately rank the free energy of the correct arrangement relative to alternative arrangements. Other authors have provided guides to small molecule docking and evaluation of the current best practices and software for this purpose [2], [3], [4], [5], [6].

Until recently, small molecule docking programs have been validated mostly on experimental structures available for the protein rather than models of the protein [7]. However, for the vast majority of protein sequences no experimental structure is available. For this reason, we and others turn our attention to evaluating small molecule docking into models of proteins [8], [9], [10], [11]. Naively, one would expect comparative models to perform better than their templates in small molecule docking as sequence deviations between template and target protein have been rectified. In particular, recent results from the Critical Assessment of Structure Prediction Techniques (CASP) indicate that comparative modeling methods can add information to models that is not present in templates [12]. However, Kairys et al. found that docking into the experimental templates performed as well as docking into the homology models based on templates with sequence identities ranging from 30% to 90% and heavy atom RMSDs in the binding site ranging from 1–4 Å [9]. On the other hand, McGovern and Shoichet found that docking into a set of comparative models covering ten enzymes from ModBase is more successful than docking in just the experimental structure of the protein with no ligand bound (apo) in a virtual screening scenario [8]. Ferrara and Jacoby found that in a virtual screen for insulin-like growth factor 1 receptor kinase ligands, homology models varied in enrichment capacity from random to as good as the experimental structure of the protein determined by X-ray crystallography [13].

Although, stunning progress has been made in *de novo* protein structure prediction over the past years [14], comparative models regularly achieve atomic detail accuracy in recent CASP experiments [14], [15], [16]. Hence, comparative modeling remains the method of choice if a template with a structure similar to the target protein can be identified in the protein data bank (PDB) [17]. Template-based modeling focuses on modifying the known structure to reflect the sequence of the protein of interest. Generally, good quality models result if the sequence identity between the target and template protein is better than 30% [18]. However, results from the latest CASP experiment indicate that template detection methods are able to identify suitable templates with even lower sequence homology [19], [20]. Only recently has high accuracy refinement of comparative models led to improvements upon the starting models [12]. Comparative models accurate at atomic detail also open the possibility of obtaining highly accurate models of protein-small molecule complexes from comparative models of proteins.

In the following experiments, we establish a baseline for the performance of RosettaLigand on small molecule docking into comparative models. Previous work has shown RosettaLigand performs as well as many other leading docking software in recognized benchmarks and in comparative studies [5], [21], [22]. RosettaLigand is a particularly attractive choice for the docking into comparative models for the following reasons: (1) In contrast to many alternative programs, RosettaLigand allows docking with complete protein and ligand flexibility. This is particularly important when docking into comparative models, as the protein backbone coordinates stem from the template and are therefore inherently inaccurate. Adjustments are expected to be necessary for optimal docking results. (2) Changes between in protein side chain identity or conformation are expected between template and target within the binding pocket. RosettaLigand's rotamer approach for sampling identity and conformation of these side chains allows for efficient simultaneous optimization of these conformations and sampling of ligand conformational space through ligand rotamers. (3) The Rosetta energy function does not focus on the protein-ligand interface but rather optimizes the energy of the entire protein-ligand complex. Therefore, an energy optimization of the entire target protein in complex with the ligand is conducted allowing for more accurate ranking of ligands. (4) RosettaLigand is built into the Rosetta modeling suite which contains all algorithms necessary for comparative modeling. Thus, a complete seamless protocol for docking into comparative models can be constructed and executed minimizing the number of software tools needed for the user.

We show that RosettaLigand can correctly identify binding modes in two sets of models. The first test composed of nine models submitted during the CASP experiment allows us to verify the method on comparative models built in a blind experiment by the best comparative modeling techniques available. We also construct a test set of 21 complexes from seven proteins with models from at least two different templates. This test set expands the test to more diverse chemotypes, examines the effect of template choice, and explores the limits of sampling and scoring methods used in RosettaLigand.

## Results and Discussion

Using two sets of comparative models we show RosettaLigand is capable of sampling and identifying native-like complexes. In the first set, models for nine targets from the 8^th^ CASP experiment which contained organic ligands were used to assess the ability of RosettaLigand to dock small molecules into comparative models constructed blindly by a variety of best-practices comparative modeling protocols. The second set of seven proteins in complex with three different ligands each expands the chemotype diversity of ligands and assesses the impact of the choice of the template as comparative models were constructed from two to five templates each. Structures of the ligands can be found in Figure S1.

Two factors are critical to the success of a small molecule docking study. First, the energy function must guide the modeling method towards native-like complexes. Second, the sampling methods must allow the method to produce native-like models.

### Native-like Binding Modes in the RosettaLigand Energy Function

**Figure 1:**
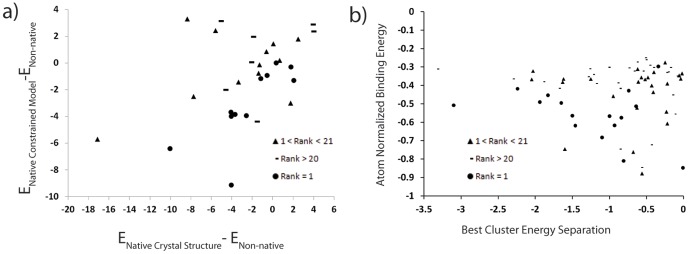
Native binding mode minima in RosettaLigand energy function. a) Energy function performance degrades in comparative models. The x-axis shows the relative depth of the native binding mode energy minima to the best score non-native binding mode found during docking. The y-axis plots the relative depth of the native binding mode in comparative models to the best scoring non-native model from docking. As expected the depth of the native binding mode energy wells decreases in comparative models. However native binding modes do not appear to score worse than non-native binding mode. b) Atom normalized binding energy and Δ Energy of the two best scoring clusters indicates the quality of docking results. A binding mode with an atom normalized binding energy of <−0.4 and a separation of <−0.5 REU is likely to be native-like. Circle native binding mode rank = 1, Triangle 1<native binding mode rank <21, Dash native binding mode rank >20.

To assess the ability of the energy function to discriminate native-like from non-native binding modes we compare native-like complexes to the lowest energy non-native conformation. We minimized native-like complexes from both the comparative models and the crystal structures and compare to non-native binding modes found in docking simulations (Table 1 and Figure 1A). Native-like complexes from relaxed crystal structures score better than non-native complexes by more than 2 Rosetta Energy Units (REUs) in 15 of the 30 cases tested. We use 2 REUs as a significant cutoff as this value corresponds approximately to 1 kcal or the strength of a weak hydrogen bond or polar interaction, i.e. the two binding modes differ in at least one such interaction [21]. In an additional 11 cases the native-like binding pose scores within 2 REUs of the best non-native binding mode. In 4 cases RosettaLigand scores non-native models significantly better than the native conformation.

**Table 1:** Minimum I-RMSD and L-RMSD of native-like binding modes.

Target PDB	# of rotatable bonds and # of atoms		Native Energy		I-RMSD		Best Non Native Docking Model				Best Native-like Docking Model			
			Cry. Str.	Model	Min.	Ave.	Energy	Rank	L-RMSD	Error	Energy	Rank	L-RMSD	I-RMSD
3D8B	6	43	−17.31	−14.94	0.74	1.28	−15.00	1	8.06	T 5.0 Å R	−9.62	263	1.57	2.09
**3DLZ**	**10**	**37**	**−17.46**	**−17.62**	**2.96**	**7.78**	**−13.78**	**4**	**6.21**		**−17.62**	**1**	**1.46**	**26.38**
3DA0	7	19	−14.16	−15.00	0.34	1.56	−17.88	1	2.84	T 1.5 Å R	−13.51	35	1.84	2.66
**3DA1**	**27**	**87**	**−28.65**	**−33.72**	**0.69**	**1.43**	**−24.59**	**2**	**14.52**		**−31.37**	**1**	**0.52**	**1.63**
3DKP	16	43	−12.3	−13.73	0.76	1.49	−16.09	1	8.59	T 6.0 Å R	−10.73	56	1.30	1.63
**3DLS**	**16**	**43**	**−12.13**	**−12.80**	**1.32**	**2.18**	**−13.92**	**2**	**4.95**		**−14.22**	**1**	**1.95**	**2.02**
*3DLC*	*11*	*51*	*−30.06*	*−24.83*	*0.90*	*4.05*	*−22.33*	*1*	*3.27*	*W*	*−20.58*	*3*	*1.40*	*2.71*
*3DME*	*27*	*87*	*−40.93*	*−29.51*	*2.43*	*3.20*	*−23.81*	*1*	*2.61*	*W*	*−21.54*	*4*	*1.04*	*3.4*
**3DOU**	**11**	**51**	**−27.92**	**−24.27**	**0.43**	**1.86**	**−17.87**	**3**	**2.66**		**−19.88**	**1**	**0.79**	**2.64**
**1Y1M**	**1**	**21**	**−13.72**	**−13.66**	**1.43**	**2.53**	**−9.68**	**14**	**2.87**		**−13.66**	**1**	**0.67**	**1.49**
*1PB9*	*0*	*14*	*−11.86*	*−9.93*	*1.09*	*2.20*	*−8.51*	*1*	*2.88*	*T 3.0 Å R*	*−7.91*	*3*	*0.86*	*2.65*
*1PBQ*	*3*	*21*	*−15.07*	*−15.78*	*1.82*	*2.50*	*−17.57*	*1*	*4.17*	*T 2.0 Å R*	*−15.78*	*8*	*1.83*	*3.48*
*2QWB*	*15*	*40*	*−10.04*	*−14.77*	*1.33*	*2.36*	*−11.78*	*1*	*4.22*	*I*	*−11.58*	*3*	*1.51*	*2.52*
*2QWD*	*11*	*39*	*−14.95*	*−14.32*	*1.31*	*2.30*	*−13.56*	*1*	*3.44*	*T 1.0 Å R*	*−12.99*	*2*	*1.46*	*1.72*
2QWE	13	44	−15.17	−17.80	1.24	2.22	−13.41	1	6.19	T 0.5 Å R	−9.93	38	1.41	1.47
1FD0	5	57	−29.62	−17.97	2.54	3.41	−21.27	1	4.45	T 3.0 Å R	−17.52	16	1.38	3.21
*1FCX*	*5*	*57*	*−26.15*	*−18.13*	*2.56*	*3.39*	*−20.57*	*1*	*5.98*	*T 5.0 Å*	*−18.13*	*10*	*1.28*	*3.27*
1FCZ	4	53	−26.14	−17.73	2.54	3.38	−20.86	1	3.19	W	−16.62	26	1.60	3.04
**1VFN**	**0**	**14**	**−11.53**	**−11.87**	**2.39**	**3.01**	**−11.87**	**1**	**6.95**	**T 5.0 Å**	**−11.87**	**1**	**1.10**	**2.39**
*1B8O*	*6*	*35*	*−16.18*	*−14.73*	*2.39*	*3.26*	*−15.58*	*1*	*4.6*	*T 3.0 Å C*	*−14.56*	*3*	*1.20*	*2.07*
**1V48**	**6**	**35**	**−19.30**	**−19.20**	**1.64**	**2.38**	**−15.21**	**3**	**3.43**		**−16.67**	**1**	**1.76**	**1.78**
2FAI	6	43	−15.38	−14.21	2.12	3.68	−14.08	1	4.08	T 3.0 Å R	−12.83	19	1.37	2.12
2AYR	10	69	−23.20	−19.12	2.68	4.33	−21.07	1	8.29	IW	−17.26	58	1.81	2.74
*2B1V*	*6*	*40*	*−14.83*	*−13.43*	*1.97*	*3.58*	*−14.88*	*1*	*3.31*	*T 2.0 Å R*	*−13.27*	*4*	*1.90*	*2.35*
**1NJA**	**6**	**33**	**−14.48**	**−14.5**	**1.75**	**3.08**	**−13.33**	**2**	**6.94**		**−14.50**	**1**	**0.66**	**3.52**
**1NJE**	**6**	**33**	**−14.90**	**−16.25**	**1.82**	**3.03**	**−12.32**	**3**	**6.01**		**−16.25**	**1**	**1.66**	**2.35**
1TSY	6	32	−16.62	−13.81	1.67	3.26	−11.81	1	6.35	T 5.0 Å R	−7.89	77	1.91	2.11
**1O3P**	**6**	**47**	**−15.88**	**−19.24**	**1.70**	**2.45**	**−17.93**	**2**	**4.84**		**−19.24**	**1**	**0.88**	**1.97**
**1F5K**	**1**	**19**	**−10.74**	**−11.11**	**1.62**	**2.23**	**−10.18**	**2**	**5.59**		**−11.11**	**1**	**0.54**	**1.62**
*1SQA*	*6*	*55*	*−19.01*	*−19.50*	*2.51*	*3.09*	*−19.69*	*1*	*2.68*	*WC*	*−17.16*	*5*	*1.66*	*2.84*

I-RMSD is calculated over all heavy atoms within 5 Å of the small molecule in X-ray crystal structure. L-RMSD is calculated over heavy atoms in the small molecule. Cluster Rank is the rank order of the cluster from lowest binding energy to highest binding energy. Error describes the spatial orientation to the native binding mode if the rank 1 cluster is non-native. I = inverted binding mode, W = wrong conformation of ligand, T = Translation in Å, R = Rotation, C = Cofactors present in native which may influence binding mode. Lines in bold indicate lowest energy binding mode is native-like. Lines in italics indicate binding mode in top 10 lowest energy modes is native like. The chemical structures can be found in Figure S1.

When analyzing the energy function with respect to complexes derived from comparative models, native-like conformations score better than non-native binding modes in 11 of 30 cases (Table 1 and Figure 1A) with a greater than 2 REUs. In 14 cases the native and non-native binding modes score within 2 REUs. A total of 5 cases display non-native binding modes that score more than 2 REUs better than the best native-like model. We conclude that inaccuracies in the comparative models negatively impact the ability of the energy function to discriminate native-like from non-native conformations – the fraction of cases where native-like models score substantially better than non-native models drops from ½ to ⅓. However, the number of cases where non-native binding modes score significantly better than native-like binding bodes stays constant at 1/6. While the energy function still recognizes native-like conformations as favorable in about 85% of the cases, its ability to discriminate non-native complexes is reduced.

We conclude the depth of the native binding mode energy well is obscured by the changes in the protein structure. Consequently we expect that multiple binding modes of similar energy are determined when docking into comparative models. Figure 1B shows a high likelihood of lowest energy structure if the atom normalized binding energy is less than −0.4 REU/atom and the separation from the next lowest energy cluster is more than −0.5 REU. An unambiguous identification of the correct binding pose from the computation alone remains difficult. However, the docking simulation will still propose a native-like binding pose as one of the low-energy conformations and can thereby guide the design of experiments to discriminate between these poses.

Multiple energetic components of the RosettaLigand energy function are necessary for proper discrimination of native-like binding modes. Previous analysis of the RosettaLigand energy function has shown the importance of hydrogen bonding and solvation energies to the correct identification of native-like binding modes [21]. In this benchmark the success of the energy function is not solely based on the van der Waals contributions, i.e. shape. When considering only the steric contributions 8 of 30 complexes contained native-like binding modes in the top ranked modes. For the remaining 13 success cases the full energy function is needed to rank a native like binding mode among the lowest energy binding modes. Figure S2 in the supplement shows the remodeling of the energy function for 1NJE which requires the full energy function in order to observe the native binding mode funnel.

The failure of the RosettaLigand energy function to unambiguously identify the native binding mode as the lowest energy binding mode in all cases is troublesome. This is likely due to inaccuracies in solvation energy. The solvation energy is an empirically derived energy function which has required manual correction when used in to design protein surfaces [23]. Thus by opening new minima up to sampling by increase the structure space sample in the comparative models it is not surprising that one would find artificially lower minima. Another point to consider is that the crystallization conditions include cofactors and impose crystal contacts that are not included in the docking calculation. So while these coordinates represent the best estimate of lowest energy conformation one should bear in mind that the energy landscape of the crystallized complex may not match the modeled conditions.

### RosettaLigand Samples Native-like Conformations at the Protein-Ligand Interface

Having looked at the energy functions ability to discriminate native-like binding poses we now turn our attention to the sampling problem. The first concern is whether Rosetta samples native-like conformations in a standard docking run. Briefly: models from 4000 independent docking simulations were filtered by energy, and submitted to clustering (see Methods). Root mean squared deviation of ligand atoms (L-RMSD), interface atoms (I-RMSD) and energy were analyzed for the lowest scoring model in each cluster. Indeed RosettaLigand samples native-like conformations (L-RMSD<2 Å) in all cases (Table 1). In the worst case (3D8B) conformations with L-RMSD<2 Å were sampled but are included in a cluster with a slightly higher L-RMSD of 2.14 Å. The lowest RMSD is listed in Table 1 for 3D8B.

**Figure 2:**
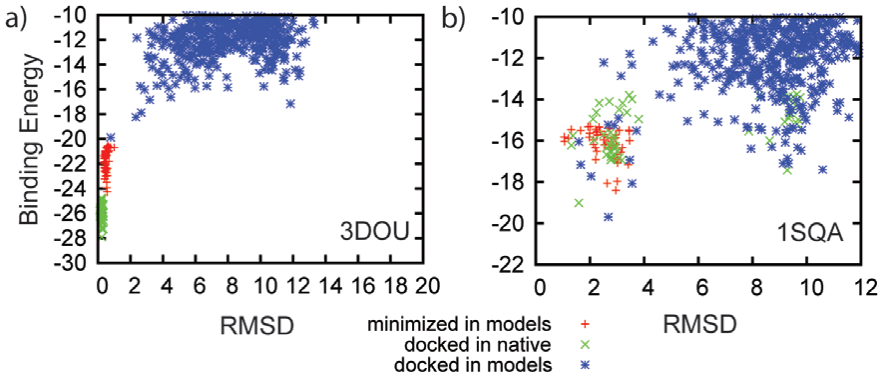
Plotting L-RMSD versus RosettaLigand binding energy displays the shape of energy wells. For target 3DOU a) the comparative model displays the global energy minimum in the same location and depth as the native binding mode. However this is not the case for all proteins as is shown in the plot for target 1SQA b). Here the energy minimum in RosettaLigand is wider, less well defined, and slightly offset between target and comparative model. Plots for all 30 targets can be found in the Figure S3. With the exception of 1SQA, 1FD0, 1FCX, 1FCZ, and 3DLS each of the other 25 targets show overlap between minima for the native binding mode minimized in the native PDB structure and the native binding mode minimized in the comparative models while L-RMSD values under 2 Å. This indicates that the scoring function generally recognizes native-like binding modes at least as local energy minimum and in 11 cases as global minimum.

Furthermore sampling in a RosettaLigand docking run is dense enough that the sampled native-like complexes fall into native energy well. For 11 of 30 cases the best energy model had a native-like binding mode with binding energy funnels like those seen in Figure 2A; 5 of these scored at least 2 REU or ∼1 kcal better than the best non-native model. A further 10 cases saw one of the top 10 best energy cluster contain a native-like binding-mode and binding energy funnels like that in Figure 2B; 8 of these scored within 2 REU of the best non-native model. In 9 cases the native-like conformations does not score as one of the top 10 choices by energy.

A lower success rate is seen if models from multiple templates are not pooled (see Tables S1, S2, S3, S4, S5, S6, and S7). On a by template basis 16 of 69 or 23% of the cases the best energy model was in a native-like binding mode, while 36 of 69 or 52% had native-like binding modes among the ten best energy clusters. We conclude that whenever possible, docking should be performed into comparative models based on multiple templates.

### Examining the Top Ranked Binding Mode Indicates Needed Improvements in Interface Refinement, Ligand Conformations, and Modeling of Cofactors

**Figure 3:**
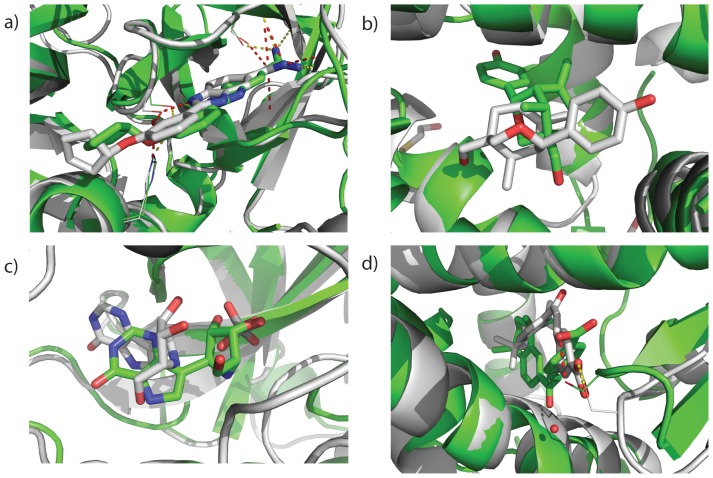
Examining low binding energy binding modes (green) in comparison to the target structure (grey) suggests avenues for improvements in ligand docking. For 10 of the 30 ligand the rank 1 ligand is a native-like binding mode as for a) target 1O3P. For 12 of the remaining cases the rank 1 binding mode is rotated and/or translated compared to the native binding mode as is seen for b) target 2FAI and c) target 1B8O. Two targets (1SQA and 1B8O) contain cofactors in the target crystal structure which overlap with the rank 1 binding mode. Panel c) shows a phosphate ion in the target crystal structure that was omitted in ligand docking. The rank 1 binding mode occupies the space of the phosphate ion. Docking results can be improved by including cofactors in the simulation. For the 6 remaining targets the rank 1 binding mode maintains many of the correct contacts but adopts a non-native conformation as seen in d) for target 1FCZ.

Encouragingly, in 11 of 30 cases the lowest energy binding mode from a docking run is the correct solution as is seen in Figure 3A for the target 1O3P. However, for 12 cases the top ranked binding mode shows deviations in translation and/or rotation similar to those seen for 2FAI and 1B8O (Figures 2B and 2C). This along with the noted decrease in native energy well depth indicates improvements are needed in the interface refinement process. This point is further illustrated in Figure 1A when plotting the difference between the depth of native energy well in the comparative models against the native energy well in the crystal structure. In this case we see a number of cases where the native energy well is much deeper but was not sampled in the comparative models (dashes and triangles with large E_nativemodel_-E_native crystal structure_ with small E_native sampled_-E_Best Non-native_). In these cases the energy function shows that ability to discriminate the native binding mode, but the docking protocol fails to sample the full depth of the native energy well given the constraints of the comparative model. In 5 cases the rank 1 binding mode maintains many of the correct interactions, but adopts a non-native conformation for the ligand as is seen in Figure 3D for 1FCZ. Further improvements in ligand conformational sampling or the energetics of ligand conformations may decrease these errors [22], [24]. However, improvements in the accuracy of the protein side chain and backbone placement will also be necessary as can be seen from the degradation of the energy well depth when comparing for crystal structures to comparative models (see Table 1). In two cases (1SQA and 1B8O) the crystallized structure contained cofactors in the binding site which overlap with rank 1 binding mode (see Figure 3C of 1B8O). Modeling the structures with these cofactors present might change the ranking of the binding modes, in fact other docking programs such as MOE already include this ability [25].

**Figure 4:**
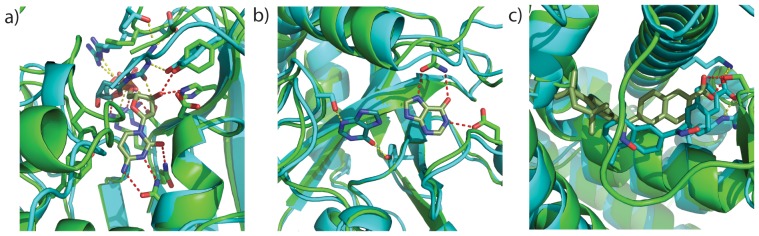
Comparison of best native and non-native binding modes. Native binding modes are green with red-dashed hydrogen bonds. Non-native binding modes are cyan with yellow-dashed hydrogen bonds. a) 1NJE Energy for the formation of hydrogen bonds discriminates the native binding mode from the best non-native binding mode. b) 1VFN Formation of hydrogen bonds is not sufficient in all cases to distinguish the native binding mode from non-native modes c) 1FD0 non-native binding modes may have deeper energy wells when forming hydrogen bonds with surface residues.

Comparing the best scoring native and non native binding modes shows well formed hydrogen bonds helps the discrimination of deep native energy wells. Figure 4A depicts 1NJE with the difference in scoring between the native and non-native binding modes of −4. The native-like binding mode shows multiple well formed hydrogen bonds. Figure 4B shows the overlay of 1VFN best native-like and non-native models which are of similar energies. The non-native binding mode forms 2 hydrogen bonds as opposed to 3 in the native binding mode however the non-native mode is able to compensate with better interactions in the other categories. Well formed hydrogen bonds assist in the discrimination of native like binding modes. However incorrect backbone placement like that seen in Figure 4C for 1FD0 can dramatically alter the energy landscape and create false minima that appear deeper than native-like binding modes. In the 1FD0 case the chance for the non-native binding mode to form incorrect hydrogen bonds obscures the true native structure. It should be noted at this point that the coordinates for the native binding mode are taken from experimental structures determined with X-ray crystallography. As such, the coordinates should be regarded as models reflecting the particular chemical environment in which the protein complex was crystallized. Thus it is possible that the minima sampled here could reflect a low energy structure in the modeled conditions.

### Careful Template Selection Improves Docking

**Figure 5:**
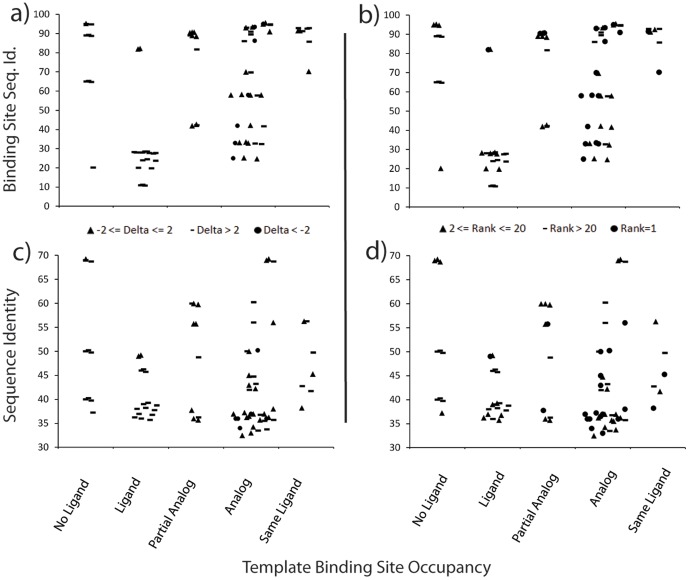
Presence of a ligand with a similar chemotype in template is more indicative of docking success than sequence identity. The figure displays docking success dependent on ligand occupation in template (x-axis) and binding site sequence identity (y-axis) in panels a) and b) and overall sequence identity in panels c) and d). Panels a) and c) classify success by energy difference (delta) between the top scoring native-like and best non-native binding mode: Circle delta <−2, Triangle −2<delta <2, Dash delta >2. Panels b) and d) determine success by rank of the best scoring native-like binding mode: Circle rank = 1, Triangle rank < = 20, Dash rank >20.

**Figure 6:**
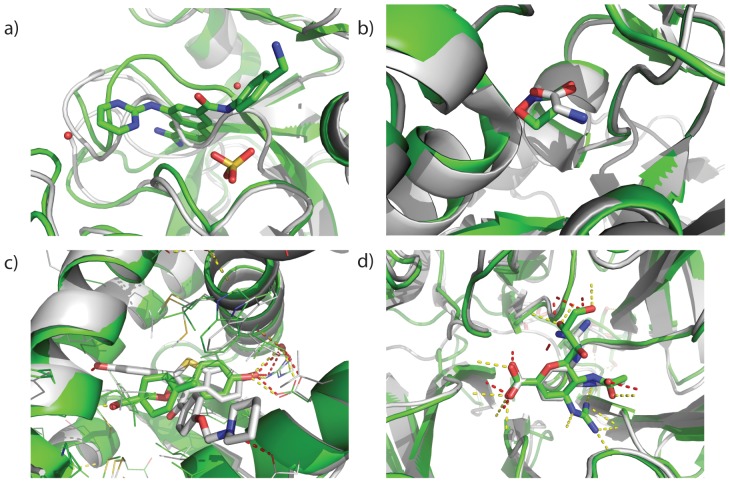
Backbone conformational differences between template and target can preclude successful docking. a) Super-imposition of 1SQA (target, green) on 1YBW (template, grey). The template has no ligand bound. Part of the template's backbone occludes the binding site. Major backbone conformational changes are needed to open the binding pocket. Selection of template with ligands similar to the target ligand pre-forms the binding site providing conserved binding motifs as seen for b) 1PB9 (target, green) on 2RC7 (template, grey), c) 2B1V (target, green) on 1QKN (template, grey), and d) 2QWE (target, green) on 1INF (template, grey). Note that the ligand analog often makes different contacts as in seen in both c) in which the phenyl group points to a different part of the pocket in the template as opposed to the target and d) in which the guanidinium head group occupies a different pocket in the binding site.

RosettaLigand has a higher success rate docking to models built from holo templates as opposed to apo structures. RosettaLigand succeeded in ∼70% of cases with templates that contain a ligand with a similar chemotype to the target ligand, in ∼50% of cases with templates with a non-similar ligand bound, and in ∼20% with templates lacking ligands). Figure 5 plots sequence identity and binding site sequence identity over template bind site occupancy. The symbols represent rank of the native-like binding mode or relative depth of the native energy minimum compared to the lowest energy non native binding mode. Success cases correlate better with ligand occupancy in the template than either sequence identity measure. Figure 6 superimposes target and template structures. Ligands in the template binding sites decrease the probability of backbone deviations occluding the native binding mode as is seen for 1SQA on 1YBW in Figure 6A. Templates with analogs in the binding site help pre-form the binding pocket, thus increasing the probability of finding native-like binding modes. Figure 6B shows almost perfect agreement between the binding mode in the target 1PB9 and for the ligand found in template 2RC7. However, target 2B1V on template 1QKN (Figure 6C) and target 2QWE on template 1INF (Figure 6D) show that not all functional groups transfer directly between complexes.

### Heuristics for Template Selection

Sequence identity of templates does not correlate with docking success. For the 21 small molecules docked into Rosetta generated models, neither the overall sequence identities nor the sequence identities in the binding site serve as a good predictor of success. This point is illustrated in Figure 5 through the lack of correlation between successful cases and sequence identity measures. At some level sequence must determine structure. Hence finer measures of sequence similarity might prove useful for example conservation of polar residues in binding pockets.

Docking into multiple templates can improve results. In 4 of 7 ligand families, docking into a comparative model based on a single template is sufficient for success (see Tables S1, S2, S3, S4, S5, S6, and S7). However a second template is needed to identify the binding mode of all three small molecules for the Neuramidase complexes (2QWE, 2QWD, 2QWB). Fan et al. also noted that multiple models improved results in the context of virtual screening [11].

Given that templates perform differently and that it is not always possible to use all available templates, a template selection heuristics would be useful. The results in this benchmark indicate that templates with sequence identities as low as 30% perform as well as or better than templates of 60%. We grouped all simulations by whether sequence similarity of template and target is below or above 50% to confirm that results are not significantly dependent on that measure. Comparing L-RMSD values we find a non-significant difference of 4.0 Å versus 3.6 Å (p-value = 0.350). Energy minimum depth also gives a non-significant difference of only 0.18 energy units (p-value = 0.394). Additionally, sequence identity in the binding site does not correlate with success.

One noticeable trend is that holo structures performed better than apo structures, particularly holo structures containing ligands similar to the target ligands. Templates containing ligands with function groups similar to the target ligand should be given preference. To test the statistical significance of the finding that docking performance depends on occupancy and type of ligand in the template, we grouped all simulations into two classes. We combined partial analogs, analogs, and identical ligands into one group. Templates with no or unrelated ligands were combined into a second. We find average L-RMSD values to be 3.7 Å versus 4.8 Å which is a significant difference with a p-value of 0.030. Comparing the relative depth of the native energy minimum to the lowest energy minimum of a non-native binding mode for the same group, the separation is larger by an average of 1.64 energy units when templates with similar ligands are used (p-value = 0.006). For the greatest gain, any chemical analogs found in the templates should be used to guide the modeling process – a finding that confirms previous research by Brylinski and Skolnick [26].

### Concluding Remarks

Modeling of small molecule protein binding sites is difficult. Davis et al. recently found that RosettaLigand and other prominent docking software failed to generate a native-like binding mode on at least one protein 70% of the time [5]. Thus docking to comparative models may seem like a fool's errand due the lack of accuracy in comparative models. However, improvements in comparative modeling techniques have increased the quality of comparative models [16], [27], [28]. Indeed in some cases the comparative models have sub-angstrom accuracy at the protein small molecule interface. The fact that, in this study, the native binding mode is sampled in all 30 cases is encouraging. Furthermore, RosettaLigand ranks the native-like binding modes from docking runs in the top 10 binding modes for 21 of 30 cases. The progressive degradation of the apparent native well energy well depth from crystal structures to comparative models indicate that docking to comparative models in Rosetta would benefit from improvements in sampling. That the docking runs into comparative models did not reach same depth of the native energy well as found during constrained minimization of the native binding mode indicates that some improvements could be gained from further refinement during the docking protocol.

We find that RosettaLigand is at least on par with alternative approaches. For example, Q-Dock reports median RMSD values of 4.4–6.0 Å for docking in comparative models of increasing difficulty [29]. RosettaLigand achieves 3.6–5.5 Å. The overall success rate of 70% is also comparable to reported values [11], [29], [30], however, comparison is complicated as benchmarks and quality measures change. A recent comparative benchmark documents that different methods have orthogonal strengths and weaknesses [31] so that application of multiple docking programs remains the recommended strategy for optimal results.

The experiments described here-in point to three improvements that could be made in sampling. First, ligands in templates could be used to guide placement of functional groups. This could be accomplished by either using the ligand placements in templates as starting positions in a small perturbation Monte Carlo minimization protocol or by implementing constraints during the docking simulation. Second, cofactors could be included in the docking process. At present, due to limitations in the code, cofactors would remain fixed in place while docking occurs; an iterative cycle could be employed to serial dock both ligands and cofactors. Upcoming changes to RosettaLigand will allow simultaneous docking of multiple ligands. Third, the homology modeling process could be altered to include ligands. Specifically, ligands found in templates could be retained in the structures during loop modeling and structure refinement as has been discussed by McCammon and others [30], [32]. This may result in more accurate comparative models and thus allow RosettaLigand to sample closer to the native binding mode.

Improvement of the scoring function is more complex. One glaring deficiency is the lack of a ligand internal energy. The internal energy of the ligand may prove particularly important for refinement of small molecule protein complexes. Examination of solvation and charge effects have also been observed to present problems for small molecule complexes [33]. Finally, using a dataset similar to this one could be used to train an artificial neural network or support vector machine classifier to pick native-like binding modes in a manner similar to that employed by NNScore, RF-Score, and FunHunt [34], [35], [36].

RosettaLigand can sample and identify native-like binding modes when docking to comparative models. Careful selection of templates and integration of biochemical data will increase the accuracy of the predicted interface. However, the native-like binding mode will be one of as many as 20 binding modes. Biochemical information will be required to prioritize the binding modes found by RosettaLigand. Once a candidate binding mode is selected it should be carefully characterized using a series of mutations. The results of mutagenesis experiments and other biochemical experiments should then be integrated in the models.

## Methods

The focus of this work was to assess the ability of RosettaLigand to identify the binding mode of small molecules using comparative models. Two sources were chosen for the comparative models. The first set of comparative models was taken from the CASP experiment. The second source of models was prepared for a subset of systems in the PDBBind, a database of small molecule-protein structures with associated binding energies. Tutorials for using Rosetta to build comparative models and also to dock small molecules have been published previously [37] and are available at the following web address http://www.meilerlab.org/index.php/jobs/resources.

### Preparation of CASP models

The models for the nine CASP targets containing organic ligands were downloaded from the CASP website (http://www.predictioncenter.org along with the corresponding crystal structures from the Protein Databank [38], [39] (www.rcsb.org). The top model submitted by each group was selected. Each model was structurally aligned to the crystal structure. First, a global alignment was performed using the PyMOL align command. This was followed by aligning all residues within 8 Å of the ligand. The ligand in the crystal structures was then transferred to the models. The procedure results in an optimally placed ligand and represents a theoretical limit for the quality models.

### Building of Comparative Models

Building of comparative models requires the selection of a structural template, alignment of the sequence onto the structural template, followed by any refinement necessary to account for changes in the structure from the new sequence. In this study, potential structural templates were identified using a blast search of sequences in the PDB. At least one template was chosen for each 10% sequence identity bin ranging from 30%–80%, if available. The templates selected for each target may be found in Tables S1, S2, S3, S4, S5, S6, and S7. A multiple sequence alignment for the selected templates was constructed using the MUSTANG structural alignment program [40]. The sequence alignment used to construct the comparative model was then created using ClustalW's sequence to profile alignment options [41]. The sequence alignment was then mapped onto the template structures.

Any gaps or insertions were remodeled using the kinematic loop closure protocol in Rosetta. Commands for the loop_model application are included in the supplemental file Methods S1. The kinematic loop closure protocol has been previously described [42]. Briefly, each loop is chosen in a random order in a Metropolis Monte Carlo protocol. Six dihedral angle torsions are chosen from the residues in the loop. The remaining torsions are randomly sampled from Ramachandran probabilities of each amino-acid. The six torsions are solved analytically. The kinematic loop closure protocol is run several hundred times over varying sections of the loop with the new conformation of the loop being accepted when it fulfills the Metropolis criteria. Once each of the loops has been built, a minimization of the protein structure is performed by iteratively performing Metropolis Monte Carlo repacking of the side chain conformations of the protein, followed by gradient minimization.

After building approximately 4000 models using the loop_model protocol, all models within 50 REU of the lowest energy models were selected for clustering. The Bio3D R package was used to align and calculate the RMSD matrix between the structures [43]. The k-means clustering algorithm in R was used to find clusters of approximately 25 members. This clustering approach is meant to pick a maximally diverse subset of the structures for docking.

**Figure 7:**
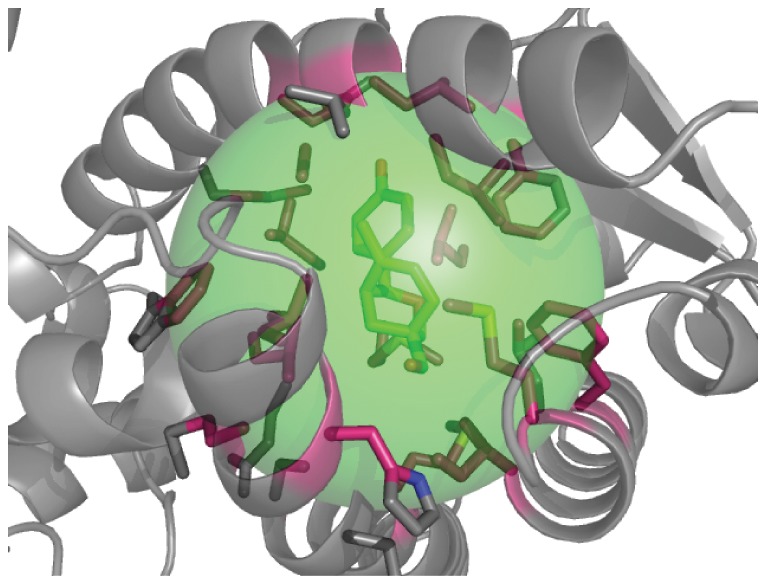
Protein small molecule interface. RosettaLigand samples complexes by randomly translating, randomly rotating, and picking a random conformation for ligand inside the green sphere. The magenta color atoms are the protein atoms included in the interface RMSD (I-RMSD) calculation.

The interface RMSD (I-RMSD) computed over non hydrogen protein atoms within 5 Å of a ligand atom in the native binding mode. The red atoms in Figure 7 give a visual representation area covered by the I-RMSD measure.

### Docking to Models

The ligand_dock application was used to dock the ligands into the protein structures. Commands for the ligand_dock, and extract_atomtree_diff application can be found in the supplemental file Methods S1. The docking protocol has been described in detail previously [22]. Ligand conformations are pre-computed for maximum rotational diversity within energetically allowed conformations. The internal ligand energy is not explicitly included in the binding energy. As only low energy conformations of the ligand are sampled, the ligand internal energy is assumed to be low and constant. Inclusion of the (small) energetic differences between ligand conformations in the binding energy calculation is a possible avenue of improvement for RosettaLigand. The protocol begins by randomly placing the ligand center of mass in a 10 Å sphere. The green sphere in Figure 7 displays the translational space covered in the docking protocol.

For the cases in the benchmark the binding site is assumed to be known. For many docking studies biological information will be available to approximate the binding pocket. In cases where the binding pocket is unknown software tools exists for the computational prediction of binding pockets [44], [45], [46].

The protocol selects 1 from up to 1000 different orientations and conformations based on shape complementarities in a low resolution van der Waals grid. The side chains and ligand in the binding site then undergo six rounds of Metropolis Monte Carlo optimization of side chain and ligand conformations. Finally, a gradient minimization yields the structure of the complex. This protocol is repeated to generate 1000 models. The models were then ordered by the interface delta score. The interface delta score is the total energy of the complex with the ligand bound minus the total energy of the complex with the ligand separated from the binding site (i.e. 500 Å from the protein).

### Identifying Binding Modes

In docking studies with non-native models, the discriminatory power of the Rosetta docking energy function is decreased [47], [48]. The global minimum of the native energy funnel may be inaccessible because of limited sampling in either the protein or the ligand. As a result, local minima cannot be distinguished from the global minimum based on the RosettaLigand energy function alone. Here, we use a clustering approach to identify the binding modes and then rank the binding modes by interface delta score. Clustering allows one to avoid considering models that contain the same binding mode.

Select best 5% of models by lowest binding energy. Compute the RMSD between the ligand heavy atoms for all pairs of models. Cluster the matrix of RMSDs in R [49] using complete linkage with a height of 3.00 RMSD. All clusters at this height are used regardless of the number of models in each cluster. The docking energy landscape is very rough. Consequently, the native binding mode may rarely be sampled. Thus, penalizing small clusters is counter-productive. Following hierarchical clustering in R, rank clusters by the energy of the best energy model in the cluster.

## Supporting Information

Figure S1 **The 2D chemical structures of the ligands docked in this benchmark show some bias towards nucleic acids.** The ligands cover a range of flexibilities from completely rigid molecules to highly flexible molecules. RosettaLigand performs better on small rigid molecules or molecules with a core fragments. Each chemical structure is label with the parent pdb code.
(TIF)

Figure S2 **The steric interactions do not fully account for the successes presented in the study.** We find that the full energy function performs better than considering just the steric components of energy function. The full energy function results in 17 successes versus just 8 when considering only the steric components.Here we compare the sum of the int_fa_atr and int_fa_rep components to the full binding energy of the complex. The red squares depict the best models as scored by the steric contributions. The blue diamonds show the best models as chosen by the full binding energy function. The full energy function scores native like models better than non-native models where as in the steric models no such preference is discernible.
(TIF)

Figure S3 **L-RMSD energy plots of complexes docked into multiple comparative models.** Blue stars display docking clusters, green crosses show top models from docking into the target structure. Red crosses show models produce by Monte Carlo minimization of native-like binding modes in the comparative models. Changes structure in comparative model vs a crystal structure alter the accessible energy landscape for RosettaLigand docking protocol. The rmsd vs energy plots for each of the test case below show the extent of the distortion in the energy landscape. Ideally cases look like the 1Y1M case were the lowest energy red, blue, and green overlap in the 0–2 Å RMSD area. Cases like 1FD0 show that if the protein structure is close to the crystal structure the algorithm scores native-like binding modes best, but that the comparative models occupy a different conformational space which favors a non-native binding mode. Finally some cases such as 1PBQ are concerning as non-native binding modes appear to score better than even native binding modes in the native crystal structure environment.
(TIF)

Methods S1 **Supplemental information for methods section including description of comparative model preparation, Rosetta commands for loop building, and Rosetta commands for ligand docking.**
(DOCX)

Table S1 **N-methyl-D-Aspartate Receptor 1 ligand docking broken down by template.** I-RMSD is calculated over all heavy atoms within 5 Å of the small molecule in X-ray crystal structure. L-RMSD are calculated over heavy atoms in the small molecule. Cluster Rank is the rank order of the cluster from lowest binding energy to highest binding energy. I = Template contains identical ligand, A = Template contains analogous ligand, PA = Template contains partial analog, L = Template contains a ligand, “-” = Template does not contain a ligand.
(DOCX)

Table S2 **Neuramidase.** ligand docking broken down by template. I-RMSD is calculated over all heavy atoms within 5 Å of the small molecule in X-ray crystal structure. L-RMSD are calculated over heavy atoms in the small molecule. Cluster Rank is the rank order of the cluster from lowest binding energy to highest binding energy. I = Template contains identical ligand, A = Template contains analogous ligand, PA = Template contains partial analog, L = Template contains a ligand, “-” = Template does not contain a ligand.
(DOCX)

Table S3 **Retanoic acid Receptor Gamma ligand docking broken down by template.** I-RMSD is calculated over all heavy atoms within 5 Å of the small molecule in X-ray crystal structure. L-RMSD are calculated over heavy atoms in the small molecule. Cluster Rank is the rank order of the cluster from lowest binding energy to highest binding energy. I = Template contains identical ligand, A = Template contains analogous ligand, PA = Template contains partial analog, L = Template contains a ligand, “-” = Template does not contain a ligand.
(DOCX)

Table S4 **Purine Nucleoside Phosphorylase ligand docking broken down by template.** I-RMSD is calculated over all heavy atoms within 5 Å of the small molecule in X-ray crystal structure. L-RMSD are calculated over heavy atoms in the small molecule. Cluster Rank is the rank order of the cluster from lowest binding energy to highest binding energy. I = Template contains identical ligand, A = Template contains analogous ligand, PA = Template contains partial analog, L = Template contains a ligand, “-” = Template does not contain a ligand.
(DOCX)

Table S5 **Estrogen Receptor ligand docking broken down by template.** I-RMSD is calculated over all heavy atoms within 5 Å of the small molecule in X-ray crystal structure. L-RMSD are calculated over heavy atoms in the small molecule. Cluster Rank is the rank order of the cluster from lowest binding energy to highest binding energy. I = Template contains identical ligand, A = Template contains analogous ligand, PA = Template contains partial analog, L = Template contains a ligand, “-” = Template does not contain a ligand.
(DOCX)

Table S6 **Thymidylate Synthase ligand docking broken down by template.** I-RMSD is calculated over all heavy atoms within 5 Å of the small molecule in X-ray crystal structure. L-RMSD are calculated over heavy atoms in the small molecule. Cluster Rank is the rank order of the cluster from lowest binding energy to highest binding energy. I = Template contains identical ligand, A = Template contains analogous ligand, PA = Template contains partial analog, L = Template contains a ligand, “-” = Template does not contain a ligand.
(DOCX)

Table S7 **Uridine Kinase Type Plasminogen Activator Ligand Docking broken down by template.** I-RMSD is calculated over all heavy atoms within 5 Å of the small molecule in X-ray crystal structure. L-RMSD are calculated over heavy atoms in the small molecule. Cluster Rank is the rank order of the cluster from lowest binding energy to highest binding energy. I = Template contains identical ligand, A = Template contains analogous ligand, PA = Template contains partial analog, L = Template contains a ligand, “-” = Template does not contain a ligand.
(DOCX)
