# Supplementary figures and images for: Using RosettaLigand for Small Molecule Docking into Comparative Models
Source: PLoS One. 2012 Dec 11;7(12):e50769. doi: 10.1371/journal.pone.0050769 (PMC3519832; doi:10.1371/journal.pone.0050769)

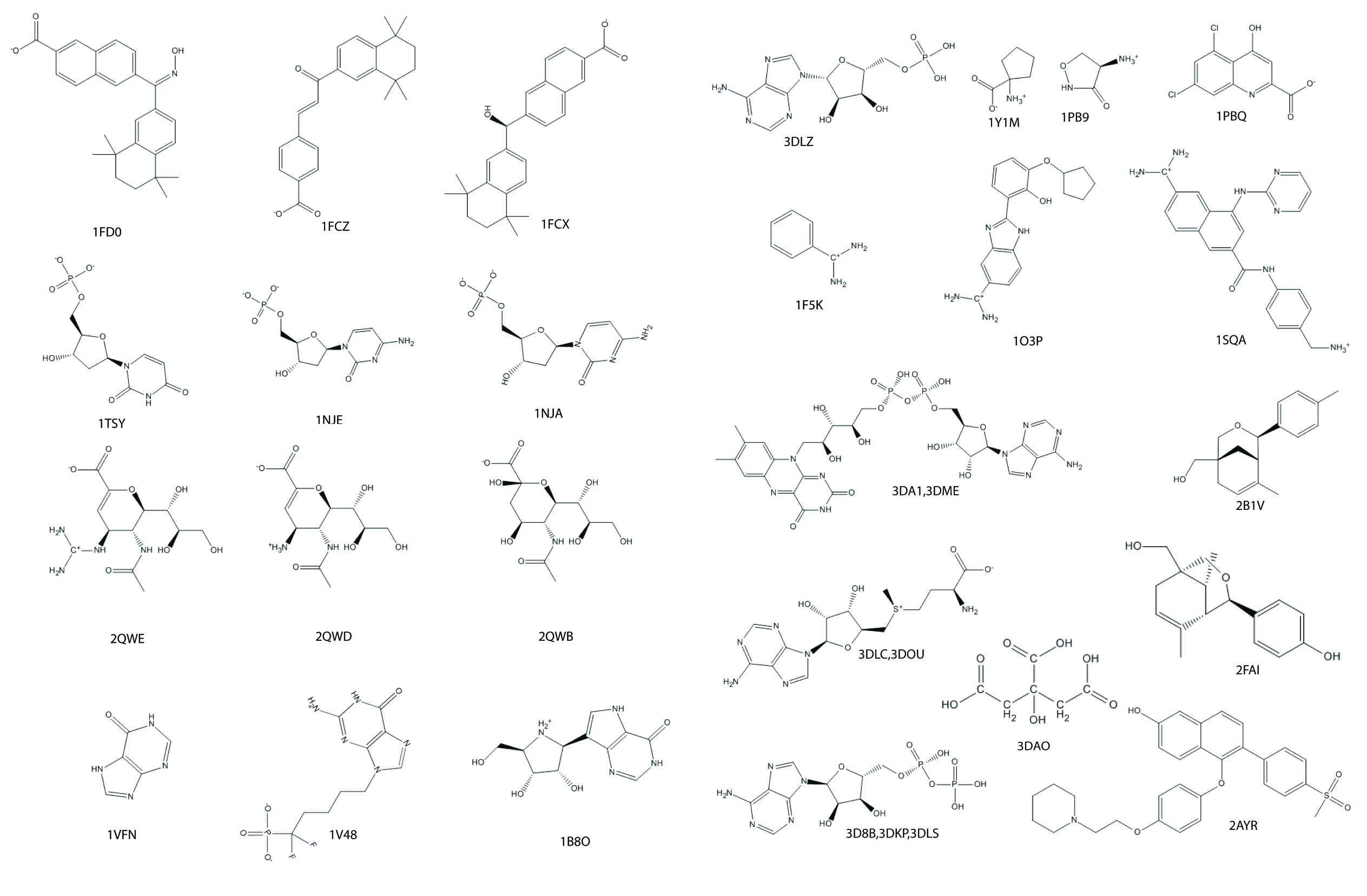

Supplement: Figure S1 — The 2D chemical structures of the ligands docked in this benchmark show some bias towards nucleic acids. The ligands cover a range of flexibilities from completely rigid molecules to highly flexible molecules. RosettaLigand performs better on small rigid molecules or molecules with a core fragments. Each chemical structure is label with the parent pdb code. (TIF) [file pone.0050769.s001.tif]

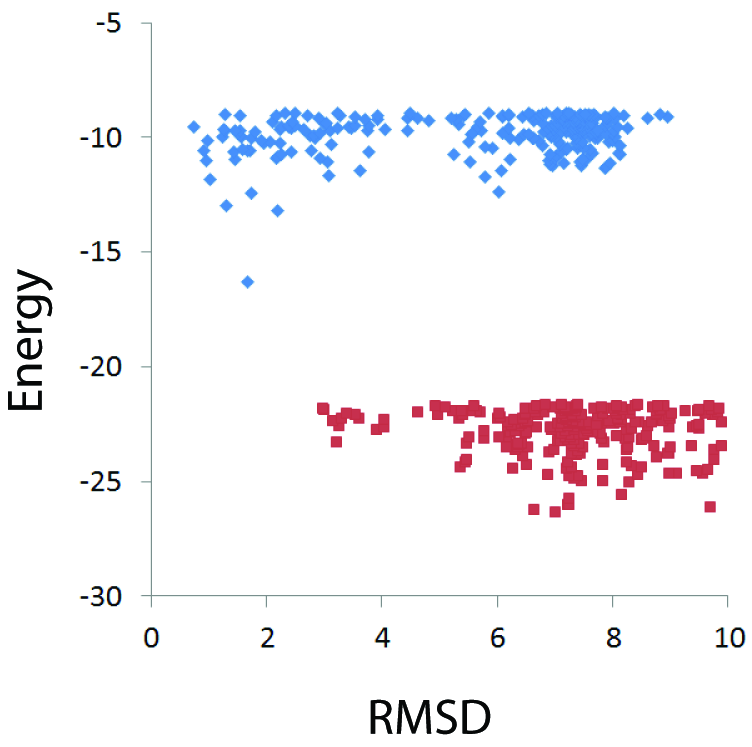

Supplement: Figure S2 — The steric interactions do not fully account for the successes presented in the study. We find that the full energy function performs better than considering just the steric components of energy function. The full energy function results in 17 successes versus just 8 when considering only the steric components.Here we compare the sum of the int_fa_atr and int_fa_rep components to the full binding energy of the complex. The red squares depict the best models as scored by the steric contributions. The blue diamonds show the best models as chosen by the full binding energy function. The full energy function scores native like models better than non-native models where as in the steric models no such preference is discernible. (TIF) [file pone.0050769.s002.tif]

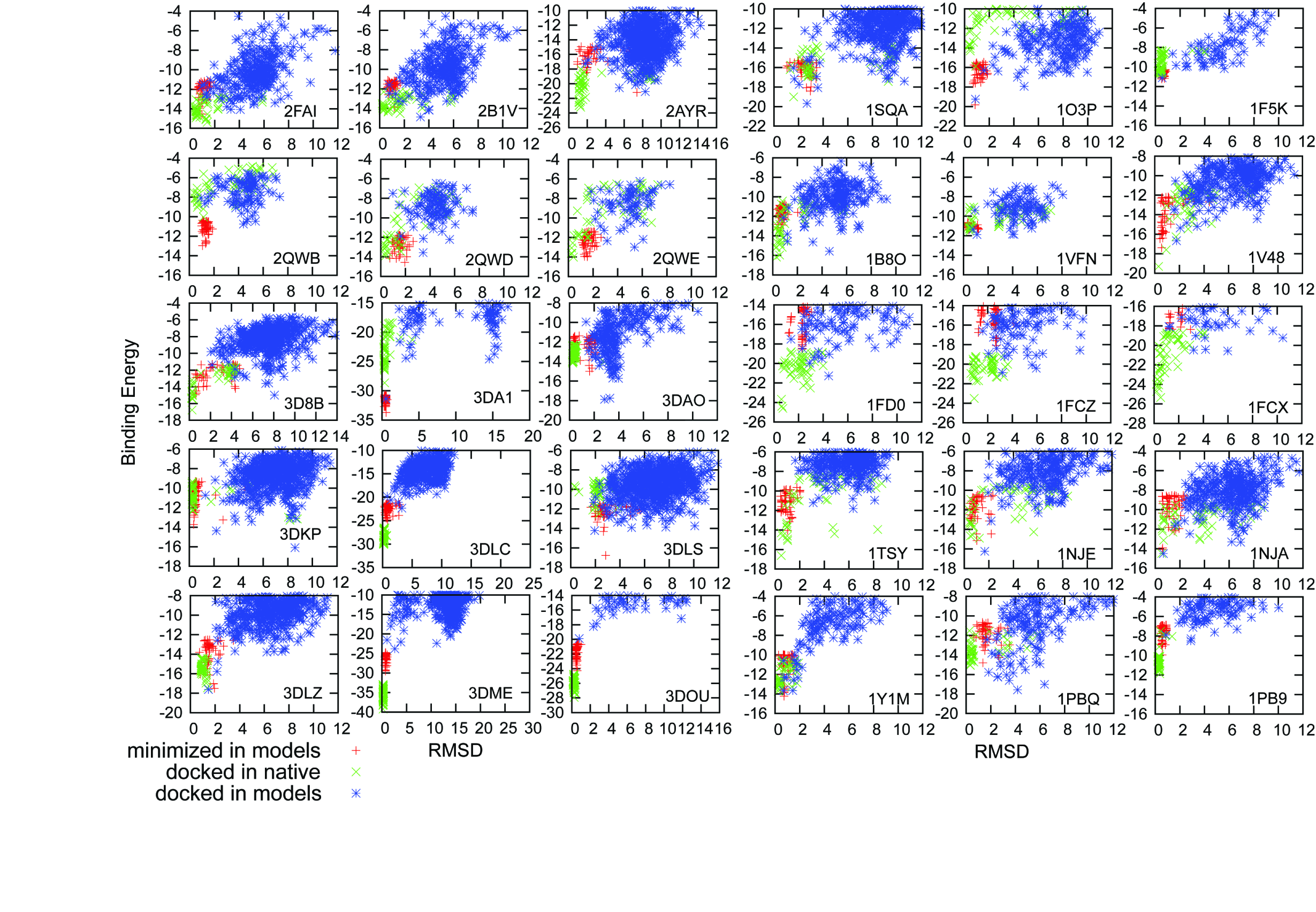

Supplement: Figure S3 — L-RMSD energy plots of complexes docked into multiple comparative models. Blue stars display docking clusters, green crosses show top models from docking into the target structure. Red crosses show models produce by Monte Carlo minimization of native-like binding modes in the comparative models. Changes structure in comparative model vs a crystal structure alter the accessible energy landscape for RosettaLigand docking protocol. The rmsd vs energy plots for each of the test case below show the extent of the distortion in the energy landscape. Ideally cases look like the 1Y1M case were the lowest energy red, blue, and green overlap in the 0–2 Å RMSD area. Cases like 1FD0 show that if the protein structure is close to the crystal structure the algorithm scores native-like binding modes best, but that the comparative models occupy a different conformational space which favors a non-native binding mode. Finally some cases such as 1PBQ are concerning as non-native binding modes appear to score better than even native binding modes in the native crystal structure environment. (TIF) [file pone.0050769.s003.tif]
