# Supplementary material for: Using RosettaLigand for Small Molecule Docking into Comparative Models
Source: PLoS One. 2012 Dec 11;7(12):e50769. doi: 10.1371/journal.pone.0050769 (PMC3519832; doi:10.1371/journal.pone.0050769)
Supplement: Methods S1 — Supplemental information for methods section including description of comparative model preparation, Rosetta commands for loop building, and Rosetta commands for ligand docking. (DOCX) [file pone.0050769.s004.docx]

**Supplemental Information for Methods Section for Using RosettaLigand for Small Molecule Docking into Comparative Models**

**Comparative Model preparation:** Target ligands were selected out of the PDBBind. Target sequences were collected from the NCBI website. Templates were selected out of 10% sequence identiy bins from a blast of PDB sequences.

blastpgp -b 250 -j 3 –h 0.001 -d pdbaa.fasta -m 9 -C psitmp.chk –i target.fasta

Template PDBs were downloaded from the RCSB.org website. Mustang Multiple Structure alignment was used to structurally align all Templates of the Target PDB. Each structural alignment was visually inspected to insure the template structure maintained the same fold as the target structure.

ClustalW profile alignment was used to align all templates to the target.

The clustalW alignment was used to thread the target sequence onto the template structures. Insertion and Deletions in the alignment were marked as loops to be rebuilt using Rosetta

Rosetta revision number 37371 loop_model application was used to rebuild the loops identified during the alignment processes. Kinematic loop closure was run with the following parameters to build the comparative models.

-in

-path

#location of rosetta_database

-database *rosetta_database_dir/*

-loops

#allow ramachandran biased sampling of backbone torsions

#under kinematic loop closer protocol

-nonpivot_torsion_sampling

#Allow expansion of loop definitions if necessary

-random_grow_loops_by 4

#build loops in random order

-random_order

#perform full atom kinematic loop closure

-refine refine_kic

#use reduced number of cycles for faster modeling

-fast

#perform iterative side chain repacking and gradient minimization

#to refine models

-relax seqrelax

#start with input pdb

-input_pdb *target.pdb*

#input file defining residues in loops

-loop_file *target.loops*

-in

-file

#Starting pdb file has side chains defined

-fullatom

-out

-file

#output models should have side chains

-fullatom

-out

#output models should be placed in models/

-path *modelsI/*

#prefix for output model pdb file names

-prefix *model_name_prefix*

#Number of models to create

-nstruct *100*

#Starting pdb file has side chains defined

-fa_input

#Use expanded chi1 and chi2 expanded rotamer sets

-ex1 -ex2

All models higher the 50 REU from the lowest energy comparative model were discarded. The remaining models were clustered by RMSD in a k-means cluster R script with k=total number of remaining models/15. The lowest energy model from each cluster was carried forward into docking.

**The ligand rotamer library** was created using the Rosetta++ small molecule conformation library generation algorithm. This algorithm samples each rotatable bond according to an empirically derived minima. After randomly assigning each rotatable bond an angle an internal energy composed of sterics salvation and hydrogen bonding is used to cull high energy conformations. After generating a set number of energetically acceptable conformations the either the lowest energy conformation of or the conformation furthest from the accepted conformation is chosen. This builds a structure maximal diverse conformation library with energetically acceptable conformation for the ligand.

**Ligand docking:** The ligand is then centered in the binding pocket and the RosettaLigand docking algiorithm is run according the parameters given below. The ligand docking algorithm randomly translates and rotates the ligand within a 10 Å sphere. It selects a random conformation from the ligand conformational library. It evaluate a coarse energy function to check for backbone clashes. If no clashes are detected, the algorithm proceeds to build in full atom sidechains and packs the sidechains and the ligand conformations in a simulated annealing of a full atom Rosetta energy function. After the 6 rounds of simulated annealing and side chain replacement. The strucuter is passed onto a gradient based full atom refinement of the binding site to move the structure to the nearest local minimum. The refinement allows some induced fit of the binding pocket through the movement of the backbone atoms in the protein.

The ligand_dock application is called with the following options

-in

-path

#location of rosetta_database

-database *rosetta_database/*

-file

#model pdb of protein with ligand placed in the binding site

-s *protein_ligand.pdb*

#Parameter file defining topology of ligand

-extra_res_fa *ligand.params*

#Native PDB of complex used as reference in RMSD calculations

-native *native_complex.pdb*

-out

#Number of models to be built

-nstruct 1000

-file

#output file for models in atom tree difference format

-silent models_compressed.out

-docking

-ligand

#allow ligand torsion angles to change during minimization

-minimize_ligand

#place a harmonic constraint with force constant of 10 on ligand torsions

#during minimization

-harmonic_torsions 10

#allow backbone phi, psi to change during minimization

-minimize_backbone

#place harmonic constraint C_α_ to start position during minimization

#with harmonic constraint of 0.3

-harmonic_Calphas 0.3

#During docking process use soften van der Waals potential

-soft_rep

#Use electrostatics potential between ligand and protein as in Rosetta 2.3

-old_estat

#Use 6 cycles of monte carlo minimization with side chain repacking

-protocol abbrev2

#attempt up to 1000 different rotations in initial placement

-improve_orientation 1000

#Use a uniform distribution to translate ligand up to five angstroms

#from starting position

-uniform_trans 5

The models are extracted from the atom tree difference silent using the extract_atomtree_diffs application with the following options

-in

-file

#Input file in atom tree difference format

-s *models_compressed.out*

#tags for models to extract from input file

-tags *model_0222*

# Parameter file defining topology of ligand

-extra_res_fa *ligand.params*

-path

#location of rosetta_database

-database *rosetta_database_dir/*

-out

#directory in which to place models

-path *models_dir/*

s
