# Supplementary material for: Using RosettaLigand for Small Molecule Docking into Comparative Models
Source: PLoS One. 2012 Dec 11;7(12):e50769. doi: 10.1371/journal.pone.0050769 (PMC3519832; doi:10.1371/journal.pone.0050769)
Supplement: Table S1 — N-methyl-D-Aspartate Receptor 1 ligand docking broken down by template. I-RMSD is calculated over all heavy atoms within 5 Å of the small molecule in X-ray crystal structure. L-RMSD are calculated over heavy atoms in the small molecule. Cluster Rank is the rank order of the cluster from lowest binding energy to highest binding energy. I = Template contains identical ligand, A = Template contains analogous ligand, PA = Template contains partial analog, L = Template contains a ligand, “-” = Template does not contain a ligand. (DOCX) [file pone.0050769.s005.docx]

| Table S1. N-methyl-D-Aspartate Receptor 1 ligand docking broken down by template. I-RMSD is calculated over all heavy atoms within 5 Å of the small molecule in X-ray crystal structure. L-RMSD are calculated over heavy atoms in the small molecule. Cluster Rank is the rank order of the cluster from lowest binding energy to highest binding energy. I=Template contains identical ligand, A=Template contains analogous ligand, PA=Template contains partial analog, L=Template contains a ligand, “-“= Template does not contain a ligand | | | | | | | | | | | | |
| --- | --- | --- | --- | --- | --- | --- | --- | --- | --- | --- | --- | --- |
| Targets | Templates | Seq.ID./  I-Seq.ID. | Crystal Structure | | I-RMSD | | Best Non-Native Binding Mode | | Model Native Binding Mode | | | |
|  |  |  | Energy | Ligand | Min | Avg. | Energy | L-RMSD | Energy | Rank | L-RMSD | I-RMSD |
| 1Y1M | 2RCA | 33%/33% |  | A | 1.57 | 2.35 | -9.68 | 2.87 | -11.51 | 1 | 1.93 | 1.68 |
|  | 2A5S | 37%/58% |  | A | 1.59 | 2.06 | -8.26 | 4.74 | -9.91 | 1 | 1.88 | 1.67 |
|  | 2I0B | 36%/25% |  | A | 1.58 | 2.46 | -8.42 | 6.72 | -11.33 | 1 | 1.91 | 1.75 |
|  | 2RC7 | 36%/33% |  | A | 1.43 | 2.31 | -9.32 | 2.75 | -13.66 | 1 | 0.67 | 1.49 |
|  | 1P1N | 34%/42% |  | A | 1.57 | 2.83 | -9.5 | 2.06 | -12.36 | 1 | 1.87 | 1.67 |
|  | Combined |  | -13.72 |  | 1.43 | 2.53 | -9.68 | 2.87 | -13.66 | 1 | 0.67 | 1.49 |
| 1PB9 | 2RCA | 33%/33% |  | A | 1.09 | 1.87 | -8.51 | 2.88 | -7.20 | 3 | 0.64 | 2.2 |
|  | 2A5S | 37%/58% |  | A | 1.10 | 1.88 | -6.54 | 3.99 | -7.18 | 1 | 0.28 | 1.14 |
|  | 2I0B | 36%/25% |  | A | 1.09 | 1.89 | -7.3 | 6.64 | -6.67 | 2 | 1.91 | 1.16 |
|  | 2RC7 | 36%/33% |  | A | 1.21 | 2.15 | -6.41 | 3.55 | -7.91 | 1 | 0.86 | 2.65 |
|  | 1P1N | 34%/42% |  | A | 1.24 | 2.55 | -7.61 | 4.81 | -7.38 | 2 | 0.46 | 1.35 |
|  | Combined |  | -11.86 |  | 1.09 | 2.20 | -8.51 | 2.88 | -7.91 | 3 | 0.86 | 2.65 |
| 1PBQ | 2RCA | 33%/33% |  | A | 1.94 | 2.47 | -14.92 | 4.46 | -9.68 | 22 | 1.88 | 2.04 |
|  | 2A5S | 37%/58% |  | A | 1.69 | 2.31 | -14.46 | 4.65 |  |  |  |  |
|  | 2I0B | 36%/25% |  | A | 1.90 | 2.49 | -14.93 | 3.59 | -13.33 | 2 | 0.88 | 2.69 |
|  | 2RC7 | 36%/33% |  | A | 1.82 | 2.49 | -15.24 | 3.65 | -12.37 | 7 | 1.73 | 2.71 |
|  | 1P1N | 34%/42% |  | A | 1.94 | 2.51 | -17.57 | 4.17 | -13.21 | 16 | 1.53 | 2.84 |
|  | Combined |  | -15.07 |  | 1.82 | 2.50 | -17.57 | 4.17 | -15.78 | 8 | 1.83 | 3.48 |
