# Supplementary material for: Using RosettaLigand for Small Molecule Docking into Comparative Models
Source: PLoS One. 2012 Dec 11;7(12):e50769. doi: 10.1371/journal.pone.0050769 (PMC3519832; doi:10.1371/journal.pone.0050769)
Supplement: Table S2 — Neuramidase. ligand docking broken down by template. I-RMSD is calculated over all heavy atoms within 5 Å of the small molecule in X-ray crystal structure. L-RMSD are calculated over heavy atoms in the small molecule. Cluster Rank is the rank order of the cluster from lowest binding energy to highest binding energy. I = Template contains identical ligand, A = Template contains analogous ligand, PA = Template contains partial analog, L = Template contains a ligand, “-” = Template does not contain a ligand. (DOCX) [file pone.0050769.s006.docx]

| Table S2. Neuramidase. ligand docking broken down by template. I-RMSD is calculated over all heavy atoms within 5 Å of the small molecule in X-ray crystal structure. L-RMSD are calculated over heavy atoms in the small molecule. Cluster Rank is the rank order of the cluster from lowest binding energy to highest binding energy. I=Template contains identical ligand, A=Template contains analogous ligand, PA=Template contains partial analog, L=Template contains a ligand, “-“= Template does not contain a ligand | | | | | | | | | | | | |
| --- | --- | --- | --- | --- | --- | --- | --- | --- | --- | --- | --- | --- |
| Targets | Templates | Seq.ID./  I-Seq.ID. | Crystal Structure | | I-RMSD | | Rank 1 |  | Model Native Binding Mode | | | |
|  |  |  | Energy | Ligand | Min | Avg. | Energy | L-RMSD | Energy | Rank | L-RMSD | I-RMSD |
| 2QWB | 2HTY | 50%/89% |  | - | 2.06 | 2.75 | -11.78 | 4.22 | -7.65 | 31 | 1.76 | 2.93 |
|  | 1V0Z | 69%/95% |  | - | 1.33 | 2.03 | -10.76 | 5.11 | -8.65 | 17 | 1.26 | 1.54 |
|  | 1INF | 36%/95% |  | A | 1.67 | 2.30 | -9.55 | 5.99 | -11.58 | 1 | 1.51 | 2.52 |
|  | Combined |  | -10.04 |  | 1.33 | 2.36 | -11.78 | 4.22 | -11.58 | 3 | 1.51 | 2.52 |
| 2QWD | 2HTY | 50%/89% |  | - | 1.89 | 2.66 | -12.96 | 5.34 |  |  |  |  |
|  | 1V0Z | 69%/95% |  | - | 1.31 | 2.01 | -13.56 | 3.44 | -12.99 | 2 | 1.46 | 1.72 |
|  | 1INF | 36%/95% |  | A | 1.67 | 2.24 | -12.22 | 5.59 | -11.88 | 2 | 1.62 | 1.81 |
|  | Combined |  | -14.95 |  | 1.31 | 2.30 | -13.56 | 3.44 | -12.99 | 2 | 1.46 | 1.72 |
| 2QWE | 2HTY | 50%/89% |  | - | 1.82 | 2.58 | -12.98 | 2.42 |  |  |  |  |
|  | 1V0Z | 69%/95% |  | - | 1.24 | 1.88 | -13.41 | 6.19 | -9.93 | 19 | 1.41 | 1.47 |
|  | 1INF | 36%/95% |  | A | 1.52 | 2.19 | -11.21 | 5.57 |  |  |  |  |
|  | Combined |  | -15.17 |  | 1.24 | 2.22 | -13.41 | 6.19 | -9.93 | 38 | 1.41 | 1.47 |
