# Supplementary material for: Using RosettaLigand for Small Molecule Docking into Comparative Models
Source: PLoS One. 2012 Dec 11;7(12):e50769. doi: 10.1371/journal.pone.0050769 (PMC3519832; doi:10.1371/journal.pone.0050769)
Supplement: Table S3 — Retanoic acid Receptor Gamma ligand docking broken down by template. I-RMSD is calculated over all heavy atoms within 5 Å of the small molecule in X-ray crystal structure. L-RMSD are calculated over heavy atoms in the small molecule. Cluster Rank is the rank order of the cluster from lowest binding energy to highest binding energy. I = Template contains identical ligand, A = Template contains analogous ligand, PA = Template contains partial analog, L = Template contains a ligand, “-” = Template does not contain a ligand. (DOCX) [file pone.0050769.s007.docx]

| Table S3. Retanoic acid Receptor Gamma ligand docking broken down by template. I-RMSD is calculated over all heavy atoms within 5 Å of the small molecule in X-ray crystal structure. L-RMSD are calculated over heavy atoms in the small molecule. Cluster Rank is the rank order of the cluster from lowest binding energy to highest binding energy. I=Template contains identical ligand, A=Template contains analogous ligand, PA=Template contains partial analog, L=Template contains a ligand, “-“= Template does not contain a ligand | | | | | | | | | | | | | | | |  |
| --- | --- | --- | --- | --- | --- | --- | --- | --- | --- | --- | --- | --- | --- | --- | --- | --- |
| Targets | Templates | Seq.ID./  I-Seq.ID. | Crystal Structure | | I-RMSD | | Rank 1 | |  | | Model Native Binding Mode | | | | |  |
|  |  |  | Energy | Ligand | | Min | Avg. | Energy | | L-RMSD | | Energy | Rank | L-RMSD | I-RMSD | |
| 1FD0 | 2ACL | 36%/28% |  | L | | 2.59 | 3.49 | -18.46 | | 2.32 | |  |  |  |  | |
|  | 1NQ0 | 37%/20% |  | L | | 2.54 | 3.28 | -19.97 | | 2.90 | | -17.52 | 5 | 1.38 | 3.21 | |
|  | 1PQ6 | 38%/28% |  | L | | 2.71 | 3.34 | -21.27 | | 4.45 | |  |  |  |  | |
|  | 2H77 | 39%/24% |  | L | | 3.22 | 3.60 | -17.40 | | 7.75 | |  |  |  |  | |
|  | Combined |  | -29.62 |  | | 2.54 | 3.41 | -21.27 | | 4.45 | | -17.52 | 16 | 1.38 | 3.21 | |
| 1FCX | 2ACL | 36%/28% |  | L | | 2.49 | 3.46 | -20.57 | | 5.98 | | -18.07 | 3 | 1.21 | 2.89 | |
|  | 1NQ0 | 37%/20% |  | L | | 2.56 | 3.3 | -20.48 | | 3.21 | | -18.13 | 7 | 1.28 | 3.27 | |
|  | 1PQ6 | 38%/28% |  | L | | 2.88 | 3.35 | -20.43 | | 4.17 | |  |  |  |  | |
|  | 2H77 | 39%/24% |  | L | | 3.12 | 3.51 | -14.01 | | 6.42 | |  |  |  |  | |
|  | Combined |  | -26.15 |  | | 2.56 | 3.39 | -20.57 | | 5.98 | | -18.13 | 10 | 1.28 | 3.27 | |
| 1FCZ | 2ACL | 36%/28% |  | L | | 2.59 | 3.49 | -19.09 | | 3.68 | | -16.62 | 7 | 1.60 | 3.04 | |
|  | 1NQ0 | 37%/20% |  | L | | 2.54 | 3.31 | -20.86 | | 3.19 | | -15.92 | 14 | 1.97 | 3.00 | |
|  | 1PQ6 | 38%/28% |  | L | | 2.71 | 3.31 | -20.08 | | 5.60 | |  |  |  |  | |
|  | 2H77 | 39%/24% |  | L | | 3.22 | 3.52 | -14.18 | | 7.11 | |  |  |  |  | |
|  | Combined |  | -26.14 |  | | 2.54 | 3.38 | -20.86 | | 3.19 | | -16.62 | 26 | 1.60 | 3.04 | |
