# Supplementary material for: Using RosettaLigand for Small Molecule Docking into Comparative Models
Source: PLoS One. 2012 Dec 11;7(12):e50769. doi: 10.1371/journal.pone.0050769 (PMC3519832; doi:10.1371/journal.pone.0050769)
Supplement: Table S4 — Purine Nucleoside Phosphorylase ligand docking broken down by template. I-RMSD is calculated over all heavy atoms within 5 Å of the small molecule in X-ray crystal structure. L-RMSD are calculated over heavy atoms in the small molecule. Cluster Rank is the rank order of the cluster from lowest binding energy to highest binding energy. I = Template contains identical ligand, A = Template contains analogous ligand, PA = Template contains partial analog, L = Template contains a ligand, “-” = Template does not contain a ligand. (DOCX) [file pone.0050769.s008.docx]

| \| Table S4. Purine Nucleoside Phosphorylase ligand docking broken down by template. I-RMSD is calculated over all heavy atoms within 5 Å of the small molecule in X-ray crystal structure. L-RMSD are calculated over heavy atoms in the small molecule. Cluster Rank is the rank order of the cluster from lowest binding energy to highest binding energy. I=Template contains identical ligand, A=Template contains analogous ligand, PA=Template contains partial analog, L=Template contains a ligand, “-“= Template does not contain a ligand \| \| \| \| \| \| \| \| \| \| \| \| \| \| --- \| --- \| --- \| --- \| --- \| --- \| --- \| --- \| --- \| --- \| --- \| --- \| --- \| \| Targets \| Templates \| Seq.ID./  I-Seq.ID. \| Crystal Structure \| \| I-RMSD \| \| Rank 1 \|  \| Model Native Binding Mode \| \| \| \| \|  \|  \| Energy \| Ligand \| Min \| Avg. \| Energy \| L-RMSD \| Energy \| Rank \| L-RMSD \| I-RMSD \| \| 1VFN \| 2P4S \| 56%/91% \|  \| A \| 2.42 \| 3.11 \| -11.84 \| 3.99 \| -9.40 \| 63 \| 0.94 \|  \| \|  \| 1G2O \| 38%/91% \|  \| A \| 2.39 \| 2.71 \| -11.87 \| 6.95 \| -11.87 \| 1 \| 1.10 \|  \| \|  \| 1TCU \| 49%/82% \|  \| L \| 2.77 \| 3.20 \| -10.53 \| 2.91 \| -11.34 \| 1 \| 0.78 \|  \| \|  \| Combined \|  \| -11.53 \|  \| 2.39 \| 3.01 \| -11.87 \| 6.95 \| -11.87 \| 1 \| 1.10 \|  \| \| 1B8O \| 2P4S \| 56%/91% \|  \| I \| 1.78 \| 2.73 \| -15.58 \| 4.60 \| -11.12 \| 16 \| 1.30 \|  \| \|  \| 1G2O \| 38%/91% \|  \| I \| 1.77 \| 2.28 \| -13.46 \| 5.53 \| -14.56 \| 1 \| 1.20 \| 2.07 \| \|  \| 1TCU \| 49%/82% \|  \| L \| 2.26 \| 2.79 \| -13.27 \| 6.89 \| -12.14 \| 6 \| 0.87 \|  \| \|  \| Combined \|  \| -16.18 \|  \| 1.77 \| 2.60 \| -15.58 \| 4.60 \| -14.56 \| 3 \| 1.20 \| 2.07 \| \| 1V48 \| 2P4S \| 56%/91% \|  \| PA \| 1.64 \| 2.34 \| -14.84 \| 2.59 \| -13.54 \| 6 \| 1.54 \|  \| \|  \| 1G2O \| 38%/91% \|  \| PA \| 1.74 \| 2.15 \| -14.73 \| 4.12 \| -16.67 \| 1 \| 1.76 \|  \| \|  \| 1TCU \| 49%/82% \|  \| PA \| 2.12 \| 2.65 \| -15.36 \| 2.07 \|  \|  \|  \|  \| \|  \| Combined \|  \| -19.30 \|  \| 1.64 \| 2.38 \| -15.36 \| 2.07 \| -16.67 \| 1 \| 1.76 \|  \| | | | | | | | | | | | | | | | |  |
| --- | --- | --- | --- | --- | --- | --- | --- | --- | --- | --- | --- | --- | --- | --- | --- | --- | --- | --- | --- | --- | --- | --- | --- | --- | --- | --- | --- | --- | --- | --- | --- | --- | --- | --- | --- | --- | --- | --- | --- | --- | --- | --- | --- | --- | --- | --- | --- | --- | --- | --- | --- | --- | --- | --- | --- | --- | --- | --- | --- | --- | --- | --- | --- | --- | --- | --- | --- | --- | --- | --- | --- | --- | --- | --- | --- | --- | --- | --- | --- | --- | --- | --- | --- | --- | --- | --- | --- | --- | --- | --- | --- | --- | --- | --- | --- | --- | --- | --- | --- | --- | --- | --- | --- | --- | --- | --- | --- | --- | --- | --- | --- | --- | --- | --- | --- | --- | --- | --- | --- | --- | --- | --- | --- | --- | --- | --- | --- | --- | --- | --- | --- | --- | --- | --- | --- | --- | --- | --- | --- | --- | --- | --- | --- | --- | --- | --- | --- | --- | --- | --- | --- | --- | --- | --- | --- | --- | --- | --- | --- | --- | --- | --- | --- | --- | --- | --- | --- | --- | --- | --- | --- | --- | --- | --- | --- | --- | --- | --- | --- | --- | --- | --- | --- | --- | --- | --- | --- | --- | --- | --- | --- | --- | --- | --- | --- | --- | --- | --- | --- | --- | --- | --- | --- | --- | --- | --- | --- | --- | --- | --- |
|  |  |  |  | |  | |  | |  | |  | | | | |  |
|  |  |  |  |  | |  |  |  | |  | |  |  |  |  | |
|  |  |  |  |  | |  |  |  | |  | |  |  |  |  | |
|  |  |  |  |  | |  |  |  | |  | |  |  |  |  | |
|  |  |  |  |  | |  |  |  | |  | |  |  |  |  | |
|  |  |  |  |  | |  |  |  | |  | |  |  |  |  | |
|  |  |  |  |  | |  |  |  | |  | |  |  |  |  | |
|  |  |  |  |  | |  |  |  | |  | |  |  |  |  | |
|  |  |  |  |  | |  |  |  | |  | |  |  |  |  | |
|  |  |  |  |  | |  |  |  | |  | |  |  |  |  | |
|  |  |  |  |  | |  |  |  | |  | |  |  |  |  | |
|  |  |  |  |  | |  |  |  | |  | |  |  |  |  | |
|  |  |  |  |  | |  |  |  | |  | |  |  |  |  | |
|  |  |  |  |  | |  |  |  | |  | |  |  |  |  | |
|  |  |  |  |  | |  |  |  | |  | |  |  |  |  | |
|  |  |  |  |  | |  |  |  | |  | |  |  |  |  | |
|  |  |  |  |  | |  |  |  | |  | |  |  |  |  | |
