# Supplementary material for: Using RosettaLigand for Small Molecule Docking into Comparative Models
Source: PLoS One. 2012 Dec 11;7(12):e50769. doi: 10.1371/journal.pone.0050769 (PMC3519832; doi:10.1371/journal.pone.0050769)
Supplement: Table S5 — Estrogen Receptor ligand docking broken down by template. I-RMSD is calculated over all heavy atoms within 5 Å of the small molecule in X-ray crystal structure. L-RMSD are calculated over heavy atoms in the small molecule. Cluster Rank is the rank order of the cluster from lowest binding energy to highest binding energy. I = Template contains identical ligand, A = Template contains analogous ligand, PA = Template contains partial analog, L = Template contains a ligand, “-” = Template does not contain a ligand. (DOCX) [file pone.0050769.s009.docx]

| Table S5. Estrogen Receptor ligand docking broken down by template. I-RMSD is calculated over all heavy atoms within 5 Å of the small molecule in X-ray crystal structure. L-RMSD are calculated over heavy atoms in the small molecule. Cluster Rank is the rank order of the cluster from lowest binding energy to highest binding energy. I=Template contains identical ligand, A=Template contains analogous ligand, PA=Template contains partial analog, L=Template contains a ligand, “-“= Template does not contain a ligand | | | | | | | | | | | | |
| --- | --- | --- | --- | --- | --- | --- | --- | --- | --- | --- | --- | --- |
| Targets | Templates | Seq.ID./  I-Seq.ID. | Crystal Structure | | I-RMSD | | Rank 1 |  | Model Native Binding Mode | | | |
|  |  |  | Energy | Ligand | Min | Avg. | Energy | L-RMSD | Energy | Rank | L-RMSD | I-RMSD |
| 2FAI | 1QKN | 60%/89% |  | PA | 2.70 | 3.26 | -14.08 | 4.08 | -11.96 | 19 | 1.66 | 2.9 |
|  | 1S9P | 36%/42% |  | PA | 2.12 | 3.94 | -13.80 | 2.98 | -12.83 | 7 | 1.37 | 2.12 |
|  | 3CS8 | 46%/11% |  | L | 2.79 | 3.83 | -13.50 | 5.16 | -11.08 | 28 | 1.66 | 3.33 |
|  | Combined |  | -15.38 |  | 2.12 | 3.68 | -14.08 | 4.08 | -12.83 | 19 | 1.37 | 2.12 |
| 2AYR | 1QKN | 60%/89% |  | A | 3.40 | 3.91 | -21.07 | 8.29 |  |  |  |  |
|  | 1S9P | 36%/42% |  | PA | 2.68 | 4.17 | -19.75 | 7.46 | -17.26 | 22 | 1.81 | 2.74 |
|  | 3CS8 | 46%/11% |  | L | 3.90 | 4.9 | -20.55 | 5.48 |  |  |  |  |
|  | Combined |  | -23.2 |  | 2.68 | 4.33 | -21.07 | 8.29 | -17.26 | 58 | 1.81 | 2.74 |
| 2B1V | 1QKN | 60%/89% |  | PA | 1.97 | 2.76 | -14.18 | 4.02 | -13.27 | 4 | 1.9 | 2.35 |
|  | 1S9P | 36%/42% |  | PA | 2.07 | 3.97 | -14.88 | 3.31 | -13.25 | 2 | 1.51 | 2.14 |
|  | 3CS8 | 46%/11% |  | L | 2.97 | 4.02 | -12.72 | 2.36 |  |  |  |  |
|  | Combined |  | -14.83 |  | 1.97 | 3.58 | -14.88 | 3.31 | -13.27 | 4 | 1.9 | 2.35 |
