# Supplementary material for: Using RosettaLigand for Small Molecule Docking into Comparative Models
Source: PLoS One. 2012 Dec 11;7(12):e50769. doi: 10.1371/journal.pone.0050769 (PMC3519832; doi:10.1371/journal.pone.0050769)
Supplement: Table S6 — Thymidylate Synthase ligand docking broken down by template. I-RMSD is calculated over all heavy atoms within 5 Å of the small molecule in X-ray crystal structure. L-RMSD are calculated over heavy atoms in the small molecule. Cluster Rank is the rank order of the cluster from lowest binding energy to highest binding energy. I = Template contains identical ligand, A = Template contains analogous ligand, PA = Template contains partial analog, L = Template contains a ligand, “-” = Template does not contain a ligand. (DOCX) [file pone.0050769.s010.docx]

| Table S6.Thymidylate Synthase ligand docking broken down by template. I-RMSD is calculated over all heavy atoms within 5 Å of the small molecule in X-ray crystal structure. L-RMSD are calculated over heavy atoms in the small molecule. Cluster Rank is the rank order of the cluster from lowest binding energy to highest binding energy. I=Template contains identical ligand, A=Template contains analogous ligand, PA=Template contains partial analog, L=Template contains a ligand, “-“= Template does not contain a ligand | | | | | | | | | | | | |
| --- | --- | --- | --- | --- | --- | --- | --- | --- | --- | --- | --- | --- |
| Targets | Templates | Seq.ID./  I-Seq.ID. | Crystal Structure | | I-RMSD | | Rank 1 |  | Model Native Binding Mode | | | |
|  |  |  | Energy | Ligand | Min | Avg. | Energy | L-RMSD | Energy | Rank | L-RMSD | I-RMSD |
| 1NJA | 1QZF | 43%/93% |  | A | 2.24 | 3.28 | -12.67 | 6.75 | -14.50 | 1 | 0.66 | 3.52 |
|  | 1J3I | 42%/93% |  | A | 2.04 | 2.92 | -13.30 | 6.21 |  |  |  |  |
|  | 1KZJ | 50%/86% |  | A | 1.75 | 3.05 | -13.33 | 6.94 | -7.21 | 103 | 1.76 | 3.06 |
|  | Combined |  | -14.48 |  | 1.75 | 3.08 | -13.33 | 6.94 | -14.50 | 1 | 0.66 | 3.52 |
| 1NJE | 1QZF | 43%/93% |  | A | 2.77 | 3.43 | -9.75 | 7.68 | -7.03 | 27 | 1.06 | 3.74 |
|  | 1J3I | 42%/93% |  | A | 1.82 | 2.66 | -11.08 | 7.15 | -9.94 | 4 | 1.23 | 1.82 |
|  | 1KZJ | 50%/86% |  | A | 1.89 | 3.01 | -13.15 | 2.18 | -16.25 | 1 | 1.66 | 2.35 |
|  | Combined |  | -14.9 |  | 1.82 | 3.03 | -12.32 | 6.01 | -16.25 | 1 | 1.66 | 2.35 |
| 1TSY | 1QZF | 43%/93% |  | I | 2.75 | 3.51 | -10.94 | 8.09 |  |  |  |  |
|  | 1J3I | 42%/93% |  | I | 1.67 | 2.68 | -11.81 | 6.35 | -7.89 | 11 | 1.91 | 2.11 |
|  | 1KZJ | 50%/86% |  | I | 2.59 | 3.60 | -10.81 | 6.32 | -7.28 | 83 | 1.24 | 3.83 |
|  | Combined |  | -16.62 |  | 1.67 | 3.26 | -11.81 | 6.35 | -7.89 | 77 | 1.91 | 2.11 |
