# Supplementary material for: Using RosettaLigand for Small Molecule Docking into Comparative Models
Source: PLoS One. 2012 Dec 11;7(12):e50769. doi: 10.1371/journal.pone.0050769 (PMC3519832; doi:10.1371/journal.pone.0050769)
Supplement: Table S7 — Uridine Kinase Type Plasminogen Activator Ligand Docking broken down by template. I-RMSD is calculated over all heavy atoms within 5 Å of the small molecule in X-ray crystal structure. L-RMSD are calculated over heavy atoms in the small molecule. Cluster Rank is the rank order of the cluster from lowest binding energy to highest binding energy. I = Template contains identical ligand, A = Template contains analogous ligand, PA = Template contains partial analog, L = Template contains a ligand, “-” = Template does not contain a ligand. (DOCX) [file pone.0050769.s011.docx]

| Table S7. Uridine Kinase Type Plasminogen Activator Ligand Docking broken down by template. I-RMSD is calculated over all heavy atoms within 5 Å of the small molecule in X-ray crystal structure. L-RMSD are calculated over heavy atoms in the small molecule. Cluster Rank is the rank order of the cluster from lowest binding energy to highest binding energy. I=Template contains identical ligand, A=Template contains analogous ligand, PA=Template contains partial analog, L=Template contains a ligand, “-“= Template does not contain a ligand | | | | | | | | | | | | |
| --- | --- | --- | --- | --- | --- | --- | --- | --- | --- | --- | --- | --- |
| Targets | Templates | Seq.ID./  I-Seq.ID. | Crystal Structure | | I-RMSD | | Rank 1 |  | Model Native Binding Mode | | | |
|  |  |  | Energy | Ligand | Min | Avg. | Energy | L-RMSD | Energy | Rank | L-RMSD | I-RMSD |
| 1O3P | 1RTF | 45%/70% |  | A | 1.70 | 2.05 | -17.99 | 4.29 | -19.24 | 1 | 0.88 | 1.97 |
|  | 1YBW | 40%/65% |  | - | 2.48 | 2.84 | -17.93 | 4.84 |  |  |  |  |
|  | Combined |  | -15.88 |  | 1.70 | 2.45 | -17.99 | 4.29 | -19.24 | 1 | 0.88 | 1.97 |
| 1F5K | 1RTF | 45%/70% |  | I | 1.62 | 1.82 | -10.18 | 5.59 | -11.11 | 1 | 0.54 | 1.62 |
|  | 1YBW | 40%/65% |  | - | 2.36 | 2.64 | -10.01 | 3.93 |  |  |  |  |
|  | Combined |  | -10.74 |  | 1.62 | 2.23 | -10.18 | 5.59 | -11.11 | 1 | 0.54 | 1.62 |
| 1SQA | 1RTF | 45%/70% |  | A | 2.51 | 2.77 | -19.69 | 2.68 | -17.16 | 4 | 1.66 | 2.84 |
|  | 1YBW | 40%/65% |  | - | 2.76 | 3.40 | -17.40 | 10.59 |  |  |  |  |
|  | Combined |  | -19.01 |  | 2.51 | 3.09 | -19.69 | 2.68 | -17.16 | 5 | 1.66 | 2.84 |
